# Chaperonins in Hepatocellular Carcinoma: Unveiling Their Role in Tumor Proliferation and Immune Modulation Through Multiomics Analysis

**DOI:** 10.1155/ijog/6152675

**Published:** 2025-11-26

**Authors:** Shou-hua Wang, Feng-ya Lv, Yuan-jie Liu, Jie-pin Li, Jia-qi Hao, Hong-hua Wang

**Affiliations:** ^1^ Department of Oncology, Xiangshui County People′s Hospital, Xiangshui, Jiangsu, China; ^2^ Department of Oncology, Affiliated Hospital of Nanjing University of Chinese Medicine, Jiangsu Province Hospital of Chinese Medicine, Nanjing, Jiangsu, China, njucm.edu.cn; ^3^ Department of General Surgery, Xiangshui County People′s Hospital, Xiangshui, Jiangsu, China

**Keywords:** cell cycle regulation, chaperonins, hepatocellular carcinoma, spatial transcriptomics, tumor-associated macrophages

## Abstract

**Background:**

Chaperonins are crucial regulators of tumor biology by controlling the stability and function of oncogenic and tumor‐suppressor proteins, influencing various tumorigenic signaling pathways. Although chaperonins have been widely discussed in various cancers, including hepatocellular carcinoma (HCC), the complex mechanisms by which they contribute to HCC progression remain insufficiently explored and require further investigation.

**Methods:**

Based on data from public databases, we screened chaperonin members from the Human Genome Organisation (HUGO) Gene Nomenclature Committee (HGNC) database. The screened genes were subjected to differential expression analysis, survival analysis, clinical correlation, and univariate Cox regression. Results were validated using single‐cell RNA (scRNA) and spatial transcriptomics (ST) data. Functional enrichment and in vitro assays were also performed.

**Results:**

Chaperonins, particularly CCT6B, were significantly overexpressed in HCC tissues, with higher expression correlating with poor prognosis in HCC patients. CCT6B was found to be involved in cell cycle regulation, promoting tumor cell proliferation. Additionally, CCT6B contributed to M2 macrophage infiltration, potentially through CCL20 signaling. Moreover, the expression levels of chaperonins were associated with *β*‐catenin activation in malignant cells, suggesting their collective involvement in HCC progression.

**Conclusion:**

This study elucidates a substantial association between dysregulated chaperonin expression profiles and genomic aberrations in pan‐cancer, underscoring the functional significance of these chaperonin molecules in understanding cell cycle regulation. Systematic characterization of chaperonin‐mediated regulatory networks enhances mechanistic insights into oncogenic processes and aberrant cellular proliferation, thereby informing the rational design of precision therapeutic interventions.

## 1. Introduction

Chaperonins are emerging as pivotal modulators of neoplastic progression, where their overexpression in tumors sustains protein homeostasis to stabilize oncoproteins and activate oncogenic signaling [1]. Paradoxically, these chaperones also function as damage‐associated molecular patterns (DAMPs), eliciting antitumor immunity while concurrently promoting cancer cell resilience against stress [2–4]. This dual role complicates therapeutic targeting, yet underscores their potential as a strategic node in combinatorial approaches. Current efforts focus on developing inhibitors that disrupt chaperonin–client interactions to destabilize protumor pathways while leveraging their immunogenic potential to enhance immunotherapy efficacy [2, 5, 6]. Elucidating context‐specific chaperonin dependencies and their interplay with the tumor microenvironment (TME) remains critical to harnessing their therapeutic promise in precision oncology.

Chaperonins facilitate the proper folding and refolding of damaged proteins to enable cancer cells to maintain functional proteome integrity, thereby mitigating endoplasmic reticulum stress and ensuring sustained proliferative capacity [7]. Emerging evidence highlights their role in modulating cell cycle checkpoints [8, 9], where chaperonin‐dependent stabilization of cyclin‐dependent kinases and mitotic regulators facilitates unscheduled cell division and tumor progression [10]. Furthermore, their ability to buffer oncogenic stress while preserving metabolic efficiency positions chaperonins as pivotal nodes linking tumor cell adaptability, proteomic stability, and unchecked proliferation in neoplastic transformation [3, 11]. While a substantial body of research has implicated chaperonins in driving malignant phenotypes across diverse tumors, their functional roles and mechanistic contributions to hepatocellular carcinoma (HCC) pathogenesis remain incompletely understood, particularly within the context of immune microenvironment modulation.

In this study, we analyzed gene expression heterogeneity and genomic variation of chaperonin molecules in pan‐cancer datasets. The results suggest that methylation and copy number variation may influence the expression of chaperonin molecules. We focused on the potential significance of chaperonins in HCC. We analyzed single‐cell sequencing and spatial transcriptome (ST) sequencing data of HCC samples to correlate chaperonin with the malignant features of the TME and performed experimental validation. It was shown that chaperonins were overexpressed in malignant cells/regions and promote malignant tumor proliferation. We also found that chaperonin is a regulator of the TME. We confirmed that the upregulation of chaperonins in HCC may be dependent on *β*‐catenin.

## 2. Materials and Methods

### 2.1. Bulk RNA‐Seq Data and Genomic Analysis

#### 2.1.1. Pan‐Cancer Analysis

Genomic variation in chaperonins was analyzed using the Genomic Cancer Analysis (GSCA) web tool. For transcriptomic data, chaperonin levels of each pan‐cancer sample were assessed using GSCA′s built‐in Gene Set Variation Analysis (GSVA) scoring method for subsequent analyses.

### 2.2. HCC‐Related Analysis

HCC‐related analysis was dependent on the Biomarker Exploration for Solid Tumors (BEST) database implementation [12].

### 2.3. Single‐Cell RNA‐Seq (scRNA‐Seq) Analysis

The scRNA‐seq dataset utilized in this study was retrieved from the TISCH2 portal (accession number GSE166635) [13]. Following initial preprocessing with the Seurat package, dimensionality reduction and cluster annotations provided by the original authors were leveraged for downstream analyses. Malignant cells were isolated and subjected to further cluster analysis. In addition, we calculated the chaperonin expression levels and cycle signaling scores using the “AddModuleScore” [14] and “CellCycleScoring” functions [15], respectively. In addition, the “scSeqComm” package [16] was used to analyze the signal exchange between different cell types.

### 2.4. ST Analysis

The ST dataset utilized in this study was retrieved from the CROST portal (accession number VISDP000078) [17]. The data were processed using the standard workflow of the “semla” package [18]. We conducted the “SPACET” algorithm [19] to evaluate the infiltration ratios of each cell type, which was visualized by the “MapFeatures” function. In particular, spatial regions dominated by malignant cells are defined as “malignant regions,” while other regions are defined as “stromal regions” or “non‐malignant regions.”

### 2.5. Gene Perturbation Analysis

To reveal potential upstream regulators of chaperonins, we retrieved gene perturbation information in GPSAdb [20].

### 2.6. Cell Culture

Human HCC cell lines Huh‐7 (#CL‐0120) and SK‐Hep‐1 (#CL‐0212) were purchased from Wuhan Puno‐sai Life Technology Co., Ltd. (Wuhan, China). The THP‐1 human monocytic cell line was obtained from the Cell Bank of the Chinese Academy of Sciences (China, #TCHu 57). THP‐1 (human monocytic leukemia cell line) cells were cultured in RPMI‐1640 medium (Gibco, United States, #31870082) supplemented with 10% fetal bovine serum (FBS) (Gibco, United States, #10099‐141). HCC cells (Huh‐7 and SK‐Hep‐1) were cultured in Dulbecco′s Modified Eagle Medium (DMEM) (Gibco, United States, #C11995500BT) supplemented with 10% FBS. All cells were maintained at 37°C in a 5% CO_2_ humidified incubator.

### 2.7. Knockdown Assay

Short interfering RNA (siRNA) targeting CCT6B was chemically synthesized as a double‐stranded siRNA duplex by Gene Chem (Shanghai, China). Three siRNA sequences (si‐CTT6B‐1, si‐CTT6B‐2, and si‐CTT6B‐3) were purchased from the supplier. Cells, at 30%–40% confluence, were transfected with these siRNA duplexes according to the manufacturer′s instructions. Knockdown efficiency was evaluated 48 h posttransfection via Western blotting (WB). The siRNA sequence exhibiting the highest knockdown efficiency (si‐CTT6B‐2) was selected for subsequent experiments, and this sequence was used for all further experiments. A nontargeting siRNA duplex was used as a negative control (NC).

si‐CTT6B‐1: 5 ^′^‐ACAACTTGTGGGCGTAGATTT‐3 ^′^ (sense) and 5 ^′^‐AAATCTACGCCCACAAGTTGT‐3 ^′^ (antisense); si‐CTT6B‐2: 5 ^′^‐GCACCCTAGAATAATAGCTGA‐3 ^′^ (sense) and 5 ^′^‐TCAGCTATTATTCTAGGGTGC‐3 ^′^ (antisense); and si‐CTT6B‐3: 5 ^′^‐GCTGCTCGATGAGATGCAAAT‐3 ^′^ (sense) and 5 ^′^‐ATTTGCATCTCATCGCAGCGC‐3 ^′^ (antisense).

### 2.8. Cell Proliferation Assay

Cell proliferation was assessed using the Cell Counting Kit‐8 (CCK‐8) assay (Beyotime Institute of Biotechnology, China, #C0038). Transfected cells (NC plasmid or si‐CTT6B plasmid) were seeded in 96‐well plates at a density of 4000 cells per well, 24 h posttransfection. Cells were cultured at 37°C in a 5% CO_2_ atmosphere. Proliferation was measured every 12 h for up to 48 h. To assess cell viability, 20 *μ*L of CCK‐8 solution was added to each well, and the cells were incubated for 2 h at 37°C. The optical density was then measured spectrophotometrically at 450 nm using a microplate reader (FLx800, BioTek, United States), according to the manufacturer′s instructions. The relative cell growth was calculated by comparing the absorbance values from each time point.

### 2.9. WB Analysis

Total protein was extracted from transfected cells by using a lysis buffer containing 150 mmol/L NaCl, 50 mmol/L Tris (pH 7.4), 1% Triton X‐100, 1% sodium deoxycholate, 0.1% SDS, and protease inhibitor cocktail (MedChemExpress, China, #HY‐K0010). Protein concentration was detected by using a bicinchoninic acid assay kit (BCA) (Beyotime Institute of Biotechnology, China, #P0010). A total of 20 *μ*g protein was added to 10% SurePAGE (GenScript, China, #M00666) and transferred to a polyvinylidene (PVDF) membrane (Millipore, United States, #IPFL00010). The membranes were blocked with blocking solution (New Cell & Molecular Biotech, China, #WB500D) for 0.5 h. Then, the membranes were incubated with a primary antibody against CTT6B (1:1000, Proteintech, United States, #67961‐1‐Ig), BBS10 (1:1000, Thermo Fisher Scientific, United States, #PA5‐100128), HSPE1 (1:1000, Proteintech, United States, #16512‐1‐AP), and GAPDH (1:5000, Proteintech, United States, #60004‐1‐Ig) at 4°C overnight. After three washes with Tris‐buffered saline containing 0.05% Tween‐20 (TBST) (Biosharp, China, #BS100), membranes were incubated with fluorescently labeled secondary antibodies (highly cross‐adsorbed goat [polyclonal] antimouse IgG[H + L] antibody [1:5000, LI‐COR, United States, #926‐68070] and highly cross‐adsorbed goat [polyclonal] antirabbit IgG[H + L] Antibody [1:5000, LI‐COR, United States, #926‐68071]) for 1 h at room temperature (22°C–25°C). Subsequently, protein bands were visualized using the LI‐COR Odyssey fluorescence imaging system (LI‐COR Biotechnology, United States), with GAPDH/*β*‐actin as loading controls for normalization.

### 2.10. Cell Cycle Analysis

Cell cycle analysis was performed using a commercial cell cycle assay kit (Beyotime Institute of Biotechnology, China, #C1052). Briefly, 1 × 10^6^ transfected HCC cells (transfected with either NC plasmid or si‐CTT6B plasmid) were fixed in 1 mL of 70% ice‐cold ethanol and incubated overnight at 4°C. Fixed cells were then washed twice with phosphate‐buffered saline (PBS), followed by incubation with 100 *μ*L of RNase A (Biosharp, China, #BS109) at 37°C for 30 min. After an additional PBS wash, the cells were resuspended in 400 *μ*L of propidium iodide (PI) staining solution and incubated for 30 min at 4°C in the dark. Cell cycle distribution was subsequently analyzed by flow cytometry.

### 2.11. Macrophage Differentiation

THP‐1 cells (1 × 10^5^ cells/well) (human monocytic leukemia cell line) were treated with phorbol 12‐myristate 13‐acetate (PMA) (MedChemExpress, China, #HY‐18739) at a concentration of 50 nM for 24 h to induce macrophage differentiation [21]. Following differentiation, the PMA‐containing medium was replaced with serum‐free medium, and the cells were incubated for an additional 24 h. HCC cells (1 × 10^5^ cells/well), either transfected with the NC plasmid or with the si‐CTT6B plasmid, were then seeded into the upper chamber of 12‐well transwell inserts (0.4‐*μ*m pore size), while the differentiated THP‐1‐derived macrophages were placed in the lower wells. After 48 h of coculture, macrophages were harvested for immunofluorescence staining.

### 2.12. Immunofluorescence Staining

The cells were then permeabilized with 0.3% Triton X‐100 (Beyotime Institute of Biotechnology, China, ST1723) in PBS for 20 min. After permeabilization, cells were washed three times with PBS. Nonspecific binding was blocked by incubating cells with 5% bovine serum albumin (BSA) (Beyotime Institute of Biotechnology, China, #ST2249) in PBS for 1 h at room temperature. The cells were then incubated overnight at 4°C with primary antibodies against CD163 (1:200, Proteintech, United States, # 68218‐1‐Ig) and CD206 (1:200, Proteintech, United States, #18704‐1‐AP). After primary antibody incubation, cells were washed three times with PBS. The cells were subsequently stained with appropriate secondary antibodies (CoraLite488‐conjugated goat antimouse IgG[H + L] [1:500, Proteintech, United States, # SA00013‐1] and CoraLite594‐conjugated goat antirabbit IgG[H + L] [1:500, Proteintech, United States, #SA00013‐4]) for 1 h at room temperature (22°C–25°C), protected from light. Following secondary antibody incubation, cells were washed three times with PBS and stained with 4,6‐diamidino‐2‐phenylindole (DAPI) (Beyotime Institute of Biotechnology, China, #C1002) for 5 min to label the nuclei. Cells were washed twice with PBS and mounted using Vectashield antifade mounting medium. Immunofluorescent images were captured using a fluorescence microscope (Olympus IX 51, Japan), with appropriate filters for detection of Alexa Fluor 488 (green) and Alexa Fluor 594 (red) signals. Nuclei were visualized based on DAPI staining.

### 2.13. Use of Artificial Intelligence Tools

No artificial intelligence or large language model (LLM) tools were used to generate, analyze, or interpret any content in this manuscript.

## 3. Results

### 3.1. Pan‐Cancer Analysis of Chaperonins

We obtained data on 15 chaperonin molecules from the Human Genome Organisation (HUGO) Gene Nomenclature Committee (HGNC) database. The 15 molecules are *BBS10*, *BBS12*, *TCP1*, *CCT2*, *CCT3*, *CCT4*, *CCT5*, *CCT6A*, *CCT6B*, *CCT7*, *CCT8*, *CLPB*, *HSPD1*, *HSPE1*, and *MKKS* (Table S1). Figure 1a illustrates the chromosomal locations of the 15 chaperonin‐coding genes (CCGs). To observe the genetic variation of CCGs in cancer, we analyze single‐nucleotide variants (SNVs) and copy number variants (CNVs) in 31 cancer types. We first examined genomic profiles and found that most CCGs were mutated at low frequencies (Figure 1b). Of the 799 patients profiled, 665 (83.23%) harbored ≥ 1 somatic mutation (Figure 1c). Mutation frequency analysis identified three genes—*BBS10*, *BBS12*, and *CCT6B*—each displaying equivalent alteration rates of 13%, a prominent feature of the cohort′s mutational landscape. Missense substitutions represented the dominant mutation class, and uterine corpus endometrial carcinoma (TCGA–UCEC) emerged as the most frequently mutated tumor histology in this cohort. This result suggests that SNV may not be a major factor influencing chaperonin expression in cancer. We further confirmed that CNV (Figure 1d) and DNA methylation (Figure 1e) are important factors affecting the expression of chaperonins. On the one hand, the expression levels of CNV and mRNA were positively correlated in most cancer types, especially in *TCP1*; on the other hand, the level of CCG methylation was negatively correlated with the level of mRNA expression in most cancers.

Figure 1Genomic and expression profiles of chaperonins in pan‐cancer analysis. (a) Chromosomal map illustrating the genomic locations of 15 chaperonin‐coding genes (CCGs). Each of the 23 human chromosomes is distinctly colored, with CCGs marked by black vertical lines labeled with gene names. (b) Heatmap of single nucleotide variant (SNV) frequencies for 15 CCGs across 31 cancer types, with color intensity ranging from light red (0% mutation frequency) to dark red (10% mutation frequency). (c) Oncoplot depicting the mutational landscape of 10 CCGs across 799 patient samples, with 665 (83.23%) harboring at least one somatic mutation. Genes are listed on the left (*BBS10*, *BBS12*, *CCT6B*, *CCT6A*, *CCT2*, *CCT3*, *CLPB*, *HSPD1*, *CCT5*, and *CCT7*), with alteration rates on the right. Mutation types are color‐coded: missense mutations in green, frameshift deletions in blue, in‐frame deletions in yellow, frameshift insertions in purple, splice site mutations in orange, nonsense mutations in red, and multihit mutations in black. Cancer types are indicated at the bottom. A tumor mutation burden (TMB) bar plot is shown at the top, with values ranging from 0 to 23 mutations per megabase (m/Mb). (d) Bubble plot showing Spearman′s correlations between copy number variants (CNVs) and mRNA expression for 15 CCGs across 31 cancer types. CCGs are listed on the left, and cancer types are at the bottom. Dot colors range from blue (Spearman′s correlation coefficient, *R* = −1) to red (*R* = 1). Dot sizes represent the −log10(FDR) values, ranging from 0 (smallest) to 100 (largest). *TCP1* shows the strongest positive correlations (*R* > 0.5, FDR < 0.05) in most cancer types, particularly in breast invasive carcinoma (BRCA). (e) Bubble plot depicting Spearman′s correlations between DNA methylation and mRNA expression for 15 CCGs across 31 cancer types. CCGs are listed on the left axis, and cancer types are along the bottom axis. (f) Box plot comparing Gene Set Variation Analysis (GSVA) scores of CCGs between tumor (red) and normal (blue) samples across 14 TCGA cancer types with > 10 paired tumor–normal samples. Most cancer types show higher GSVA scores in tumor samples (*p* < 0.05, Wilcoxon′s signed‐rank test), except for KIRC and THCA, where normal samples exhibit higher scores (*p* < 0.05). (g) Box plot depicting GSVA scores of CCGs across subtypes of selected cancer types. Subtypes are labeled below each cancer type. (h) Forest plot from univariate Cox proportional hazards regression showing survival differences between high and low Gene Set Variation Analysis (GSVA) score groups across 25 cancer types. Cancer types are listed on the left, while survival endpoints are displayed on the right, including overall survival (OS), progression‐free survival (PFS), disease‐specific survival (DSS), and disease‐free interval (DFI). Hazard ratios (HRs) are represented by dots, with color intensity ranging from white (HR = 1) to red (HR = 10) based on risk; dot sizes reflect −log10(FDR) values, ranging from 1 (smallest) to 3 (largest). CCGs are unfavorable prognostic factors (HR > 1, *p* < 0.05) in BRCA, head and neck squamous cell carcinoma (HNSC), liver hepatocellular carcinoma (LIHC), and lung adenocarcinoma (LUAD). (i) Heatmap depicting Spearman′s correlations between CCG expression and cancer‐related pathways (apoptosis, cell cycle, DNA damage, EMT, hormone AR, hormone ER, PI3K/AKT, RAS/MAPK, RTK, and TSC/mTOR) across 31 cancer types. Cancer types are listed on the left, and pathways are at the bottom. Color intensity ranges from blue (negative correlation, *R* = −0.3) to red (positive correlation, *R* = 0.6). Significant correlations are marked with  ^∗^ (*p* < 0.05) and # (FDR < 0.05). Strong positive correlations (*R* > 0.3, FDR < 0.05) are observed between CCGs and cell cycle/DNA damage pathways in most cancer types. (j, k) Scatter plots showing Pearson′s correlations between CCG expression and cell cycle scores across pan‐cancer samples. (j) The plot depicts G2/M phase correlations (*R* = 0.61, *p* < 0.001), and (k) the plot depicts S phase correlations (*R* = 0.66, *p* < 0.001). Dots are colored black, with sizes reflecting sample density (ranging from 1 to 5). Data were analyzed using the GEPIA web tool.

**Figure:**
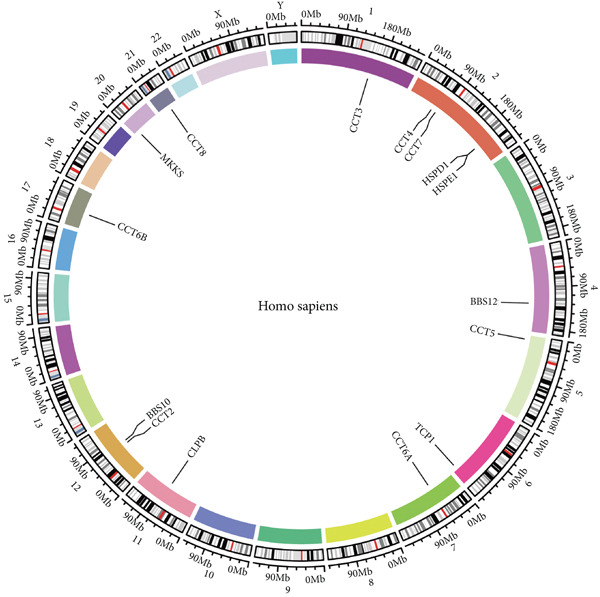
(a)

**Figure:**
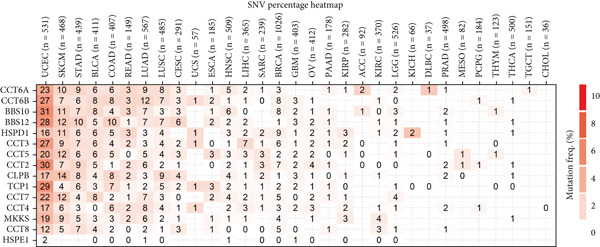
(b)

**Figure:**
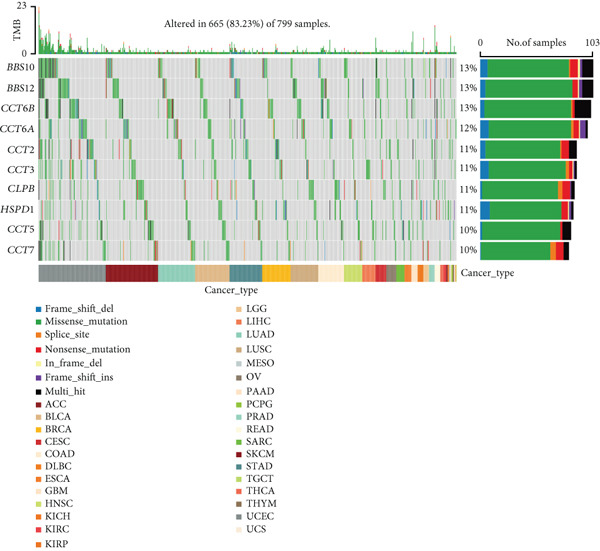
(c)

**Figure:**
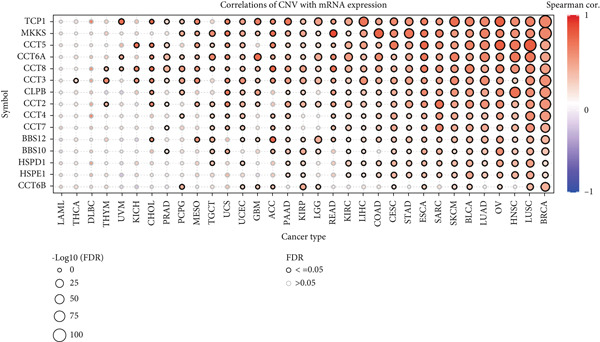
(d)

**Figure:**
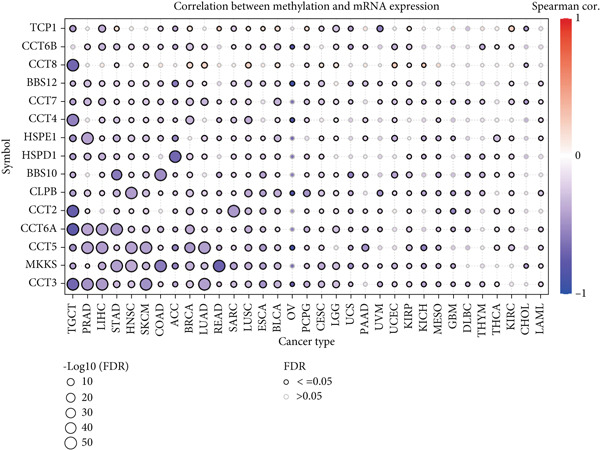
(e)

**Figure:**
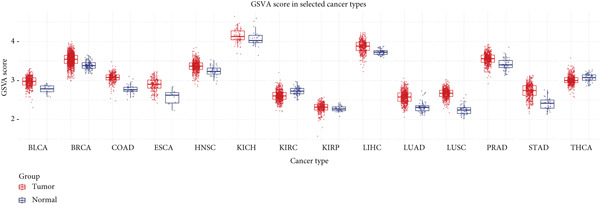
(f)

**Figure:**
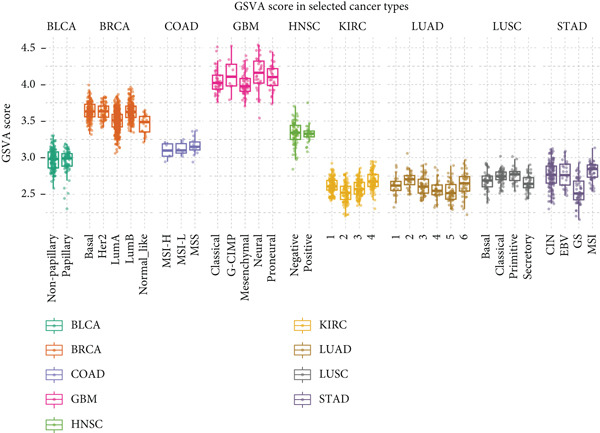
(g)

**Figure:**
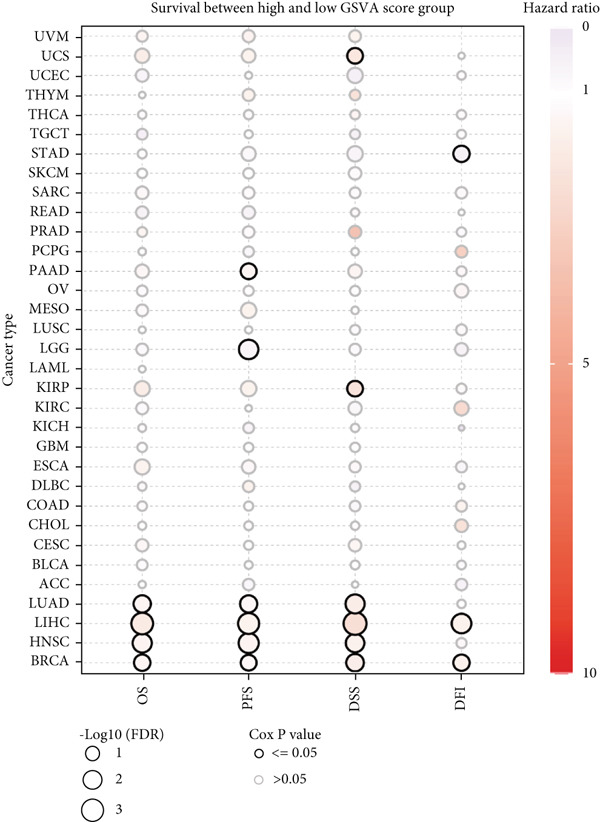
(h)

**Figure:**
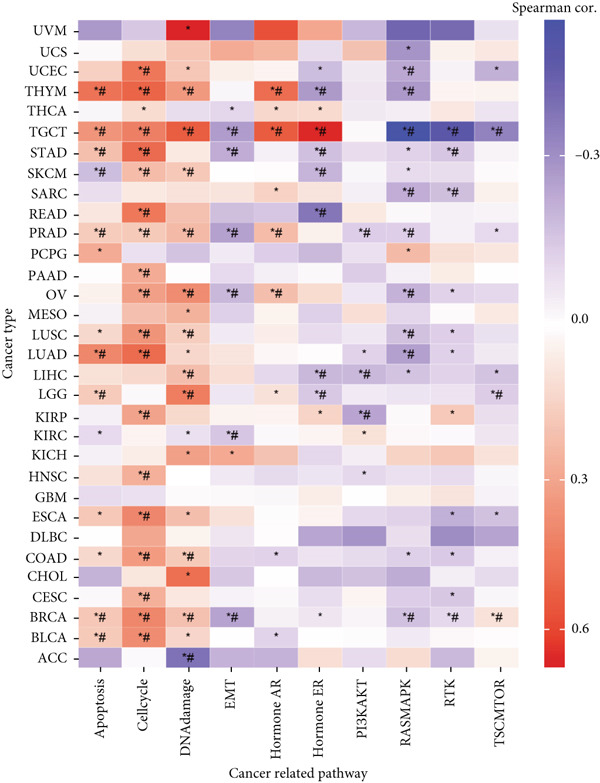
(i)

**Figure:**
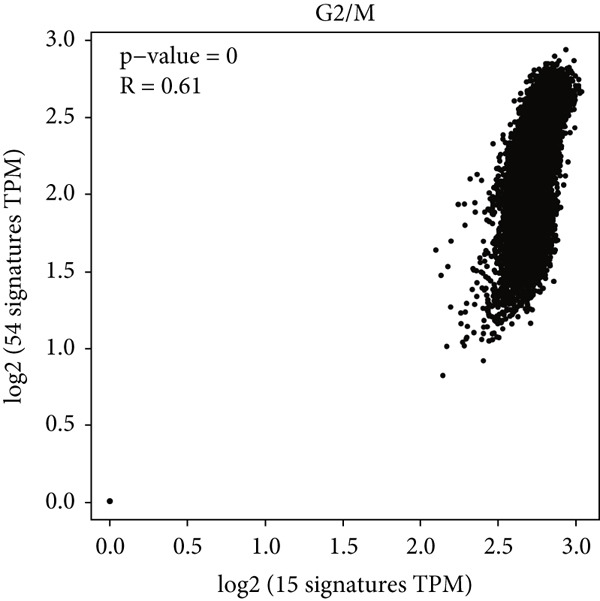
(j)

**Figure:**
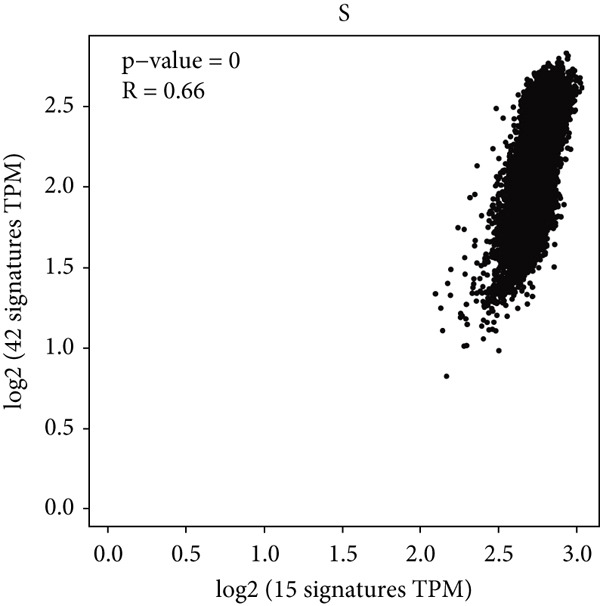
(k)

We then analyzed the difference in CCG expression between normal and cancer samples. The CCG expressions were based on the enrichment score calculated by GSVA. Since only 14 TCGA cancer types contained > 10 paired tumor–normal samples, we conducted mRNA differential expression analysis using these matched datasets to ensure statistically robust results. As shown in Figure 1f, with the exception of kidney renal clear cell carcinoma (KIRC) and thyroid carcinoma (THCA), the majority of CCGs were upregulated in tumor tissues. We further analyzed the difference in CCG expression among different subtypes of different malignancies and identified the correlations (Figure 1g). For instance, CCGs showed the highest expression in microsatellite instability (MSI)–type gastric cancer. To evaluate the prognostic implications of CCGs in cancer patients, we implemented a univariate Cox proportional hazards regression model, with overall survival (OS), progression‐free survival, disease‐specific survival (DSS), and disease‐free interval designated as the primary clinical endpoints. As in Figure 1h, we observed that CCGs were unfavorable factors in breast invasive carcinoma (BRCA), head and neck squamous cell carcinoma (HNSC), liver hepatocellular carcinoma (LIHC), and lung adenocarcinoma (LUAD).

We next explored the characterization of chaperonin‐related signaling pathways. In most cancer types, chaperonins showed a high level of activation in the “CellCycle” and “DNADamage” signaling pathway (Figure 1i). To validate this result, we calculated the relationship between CCGs and cell cycle scores in pan‐cancer data and found a highly positive correlation between CCGs and G2/M (Figure 1j) phase, as well as S phase (Figure 1k). This aligns with prior research, reinforcing the validity of our results.

### 3.2. Expression and Prognostic Analysis of Chaperonins in HCC

Since chaperonins show significant prognostic value in LIHC, we focus further on LIHC here. To ensure the reliability of our results, we included multiple HCC datasets for subsequent analyses. As shown in Figure 2a, there were high expressions of CCGs in the HCC tumor samples compared with the normal controls (*p* < 0.05). To evaluate chaperonin protein expression levels, we analyzed immunohistochemical (IHC) staining data from the Human Protein Atlas (HPA). Figure 2b demonstrates significantly elevated chaperonin expression in cancerous tissues relative to normal controls. In the examination of the clinical parameters’ relevance, we discovered that chaperonin expression was associated with tumor grade, microvascular invasion (MVI0), alpha‐fetoprotein (AFP), response to sorafenib, and Hepatitis B virus (HBV) infection (Figure 2c). Importantly, chaperonins are associated with poor prognosis in HCC, including OS, DFS, DSS, and PFS (Figure 2d). These results indicated that chaperonins may play an important role in the clinical progression of HCC. Pathologic sections suggest that samples with high‐chaperonin expression may have a richer stromal composition (Figure 2e). Furthermore, TCGA–LIHC‐based correlation analysis showed a positive correlation between CCGs (Figure 2f), implying that chaperonins may synergize to perform biological functions.

Figure 2Expression and prognostic significance of chaperonins in hepatocellular carcinoma (HCC). (a) Violin plots comparing mRNA expression levels of CCGs between normal (blue) and HCC tumor (yellow) samples across five datasets. The *y*‐axis represents chaperonin expression (log2‐transformed). Tumor samples show significantly higher CCG expression compared to normal controls (Wilcoxon′s test, *p* values: E‐TABM‐36, *p* = 0.001; GSE144269, *p* = 2.7e − 06; GSE14520, *p* < 2.2e − 16; GSE54236, *p* = 0.00059; and TCGA–LIHC, *p* < 2.2e − 16). (b) Representative immunohistochemical (IHC) staining images from the Human Protein Atlas (HPA) showing protein expression of 15 CCGs in HCC tumor tissues (T) versus normal liver tissues (N). Tumor tissues exhibit darker brown staining (indicating higher expression) compared to normal tissues with minimal staining. (c) Violin plots depicting chaperonin expression across clinical parameters in HCC: tumor grade, microvascular invasion (MVI), alpha‐fetoprotein (AFP) levels, sorafenib response, and Hepatitis B virus (HBV) infection. (d) Forest plot from univariate Cox regression analysis showing hazard ratios (HRs) for survival outcomes in HCC across multiple datasets: OS, DFS, RFS, DSS, and PFS. (e) Representative hematoxylin and eosin (HE)–stained sections of HCC tissues with low and high CCG expression. Low‐magnification views (left; scale bars, 500 *μ*m) show overall tissue morphology, while high‐magnification images (right; scale bars, 20 *μ*m) demonstrate increased stromal content in high‐expression samples. (f) Circular correlation plot (chord diagram) showing Pearson′s correlations between CCGs based on the TCGA–LIHC data. CCGs are arranged around the circle, with color intensity ranging from blue (*R* = −1) to red (*R* = 1). Positive correlations are observed among most CCGs, suggesting synergistic biological functions.

**Figure:**
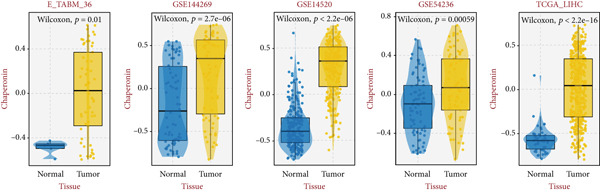
(a)

**Figure:**
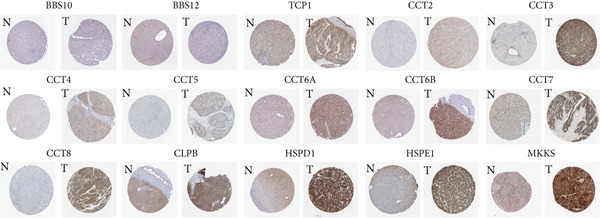
(b)

**Figure:**
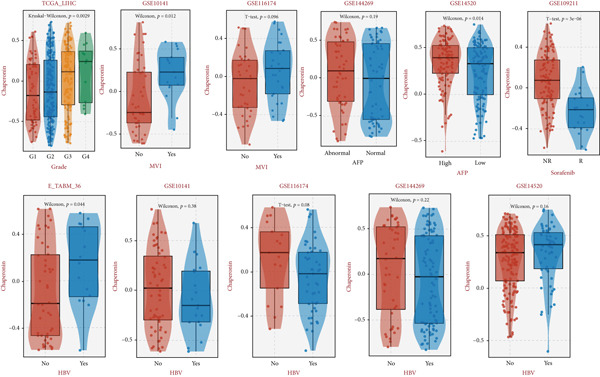
(c)

**Figure:**
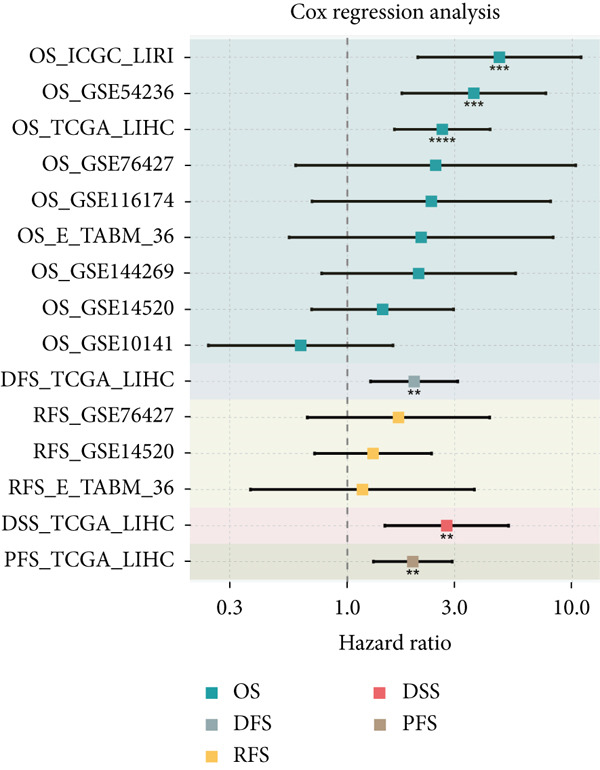
(d)

**Figure:**
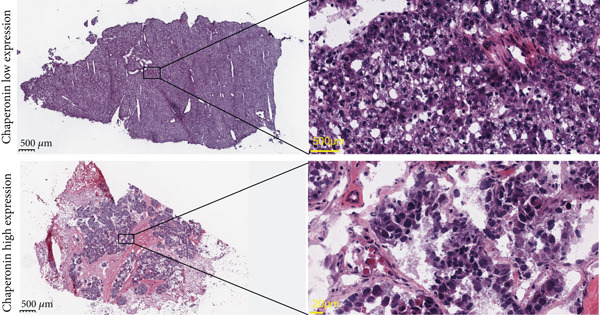
(e)

**Figure:**
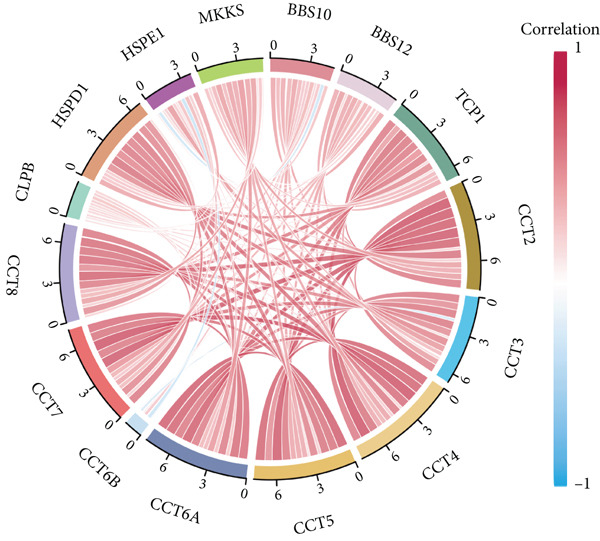
(f)

### 3.3. Chaperonins Are Upregulated in Malignant Cells and Malignant Regions

The rise of single‐cell and ST sequencing technologies has allowed researchers to dissect gene expression patterns at a high resolution. To characterize chaperonin expression within the TME, we first analyzed scRNA‐seq data from HCC samples. Figures 3a, 3b, and 3c outline the single‐cell analysis pipeline, including a Uniform Manifold Approximation and Projection (UMAP) visualization, a bar plot of cell type distributions, and a bubble plot for cluster‐specific marker expression. The expression level of CCGs was calculated with the “AddModuleScore” function and is illustrated in Figure 3d. The UMAP plot indicated that CCGs are upregulated in malignant cells. In addition to evidence from scRNA‐seq, we also utilized ST data, including four normal samples, four tumor edge samples, one portal vein tumor thrombus sample, and four tumor samples. For each sample, clustering was performed using the “BayesSpace” package [22] to identify characteristic regions. Based on inferred CNV profiles, each spot was classified into distinct categories: tumor region, interface region, or stromal region. Similar to the results of scRNA‐seq, CCGs were expressed at low levels in normal samples and significantly upregulated in malignant regions (Figures 3e, 3f, 3g, and 3h). Together, the results suggest that chaperonins may function in TME primarily through tumor cells.

Figure 3Chaperonin expression patterns in HCC malignant cells and tumor microenvironment. (a) Uniform Manifold Approximation and Projection (UMAP) plot showing dimensionality reduction of HCC cells based on gene expression profiles, with cell clusters color‐coded by cell type (malignant, immune, stromal, and others). (b) Bar plot illustrating the relative proportions of cell types within the scRNA‐seq dataset. (c) Heatmap illustrating expression levels of cluster‐specific marker genes across cell types, with rows representing cells and columns representing genes. (d) UMAP plot displaying chaperonin expression scores (calculated using the Seurat “AddModuleScore” function), revealing elevated chaperonin expression in malignant cell clusters compared to nonmalignant populations. (e–h) Spatial transcriptomic analysis of HCC samples (normal, tumor edge, portal vein tumor thrombus, and tumor). Yellow color‐coded spots reveal chaperonin expression predominantly enriched in tumor regions.

**Figure:**
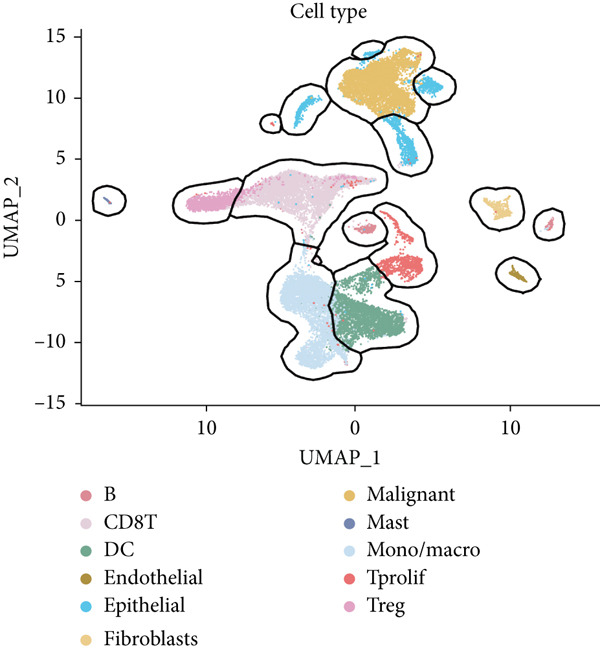
(a)

**Figure:**
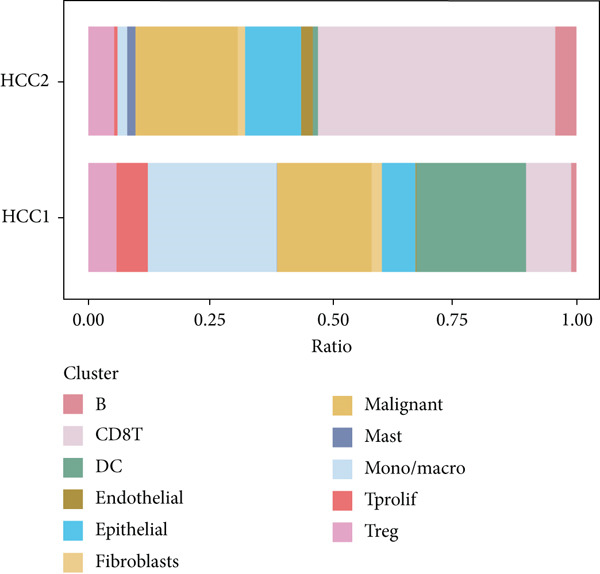
(b)

**Figure:**
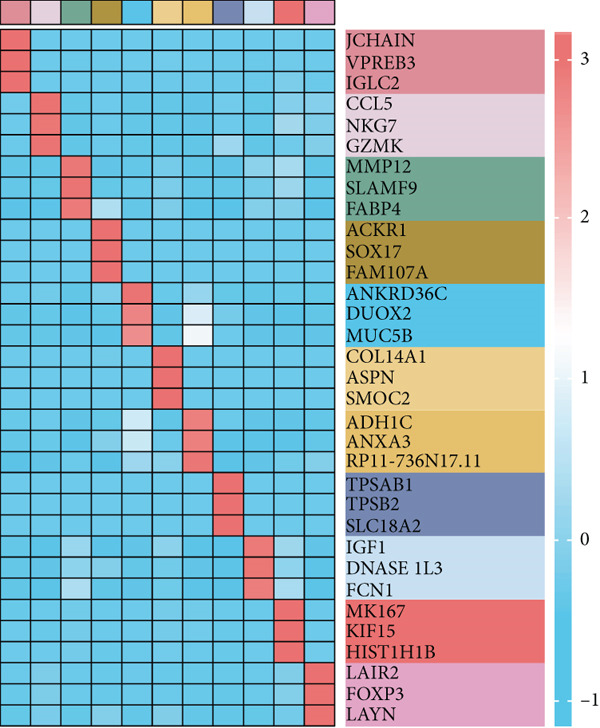
(c)

**Figure:**
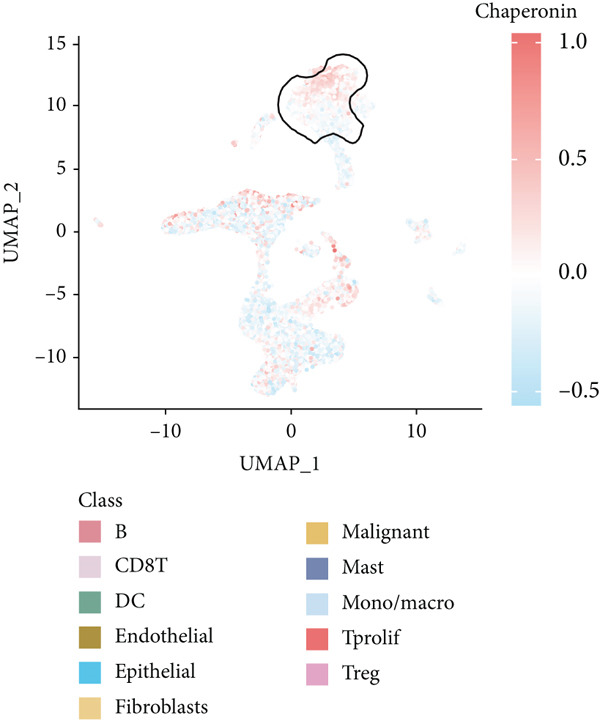
(d)

**Figure:**
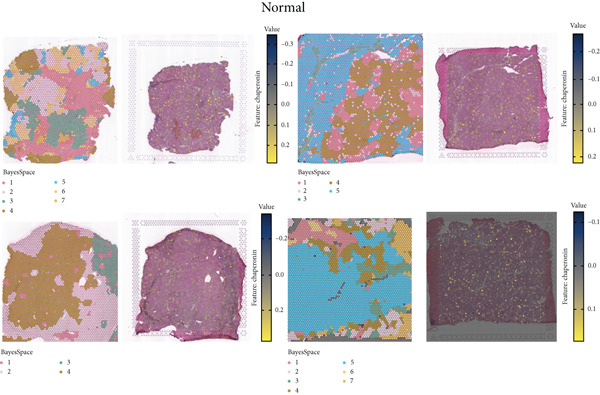
(e)

**Figure:**
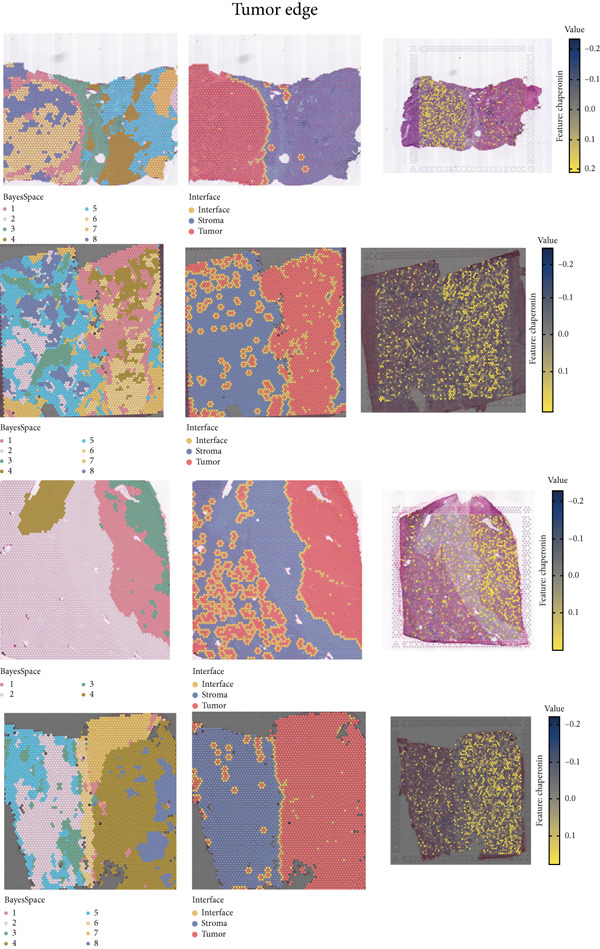
(f)

**Figure:**
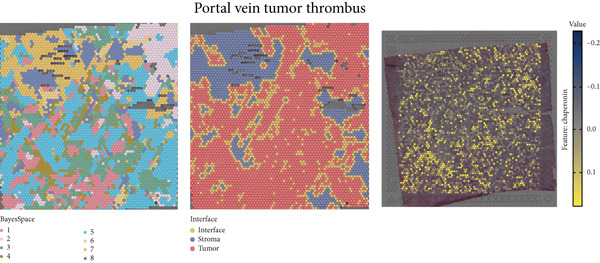
(g)

**Figure:**
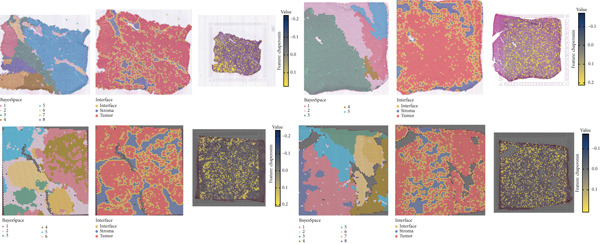
(h)

### 3.4. Chaperonins Are Involved in Cell Cycle Regulation

Since chaperonins are upregulated in malignant cells, we next sought to investigate their biological function. As shown in Figure 4a, the top 20 related genes of CCGs were identified using the GeneMinia online tool. Among these genes, we observed a large number of cancer‐related genes, such as *ACTB* (encoding *β*‐actin), *IMMT* (encoding inner membrane mitochondrial protein), and PFDNs (encoding Prefoldin). Functional enrichment analyses of chaperonins were performed using the BEST database. As expected, chaperonins may be involved in a substantial number of signaling pathways, especially cell cycle–related pathways (Figure 4b,c). In order to seek further evidence, we conducted clustering analysis of malignant cells and revealed three subclusters. As shown in Figures 4d, 4e, and 4f, malignant cells with high expression of CCGs were enriched with a large variety of cell cycle–related genes. In the ST data from four tumor specimens, CCGs were also observed to be highly expressed in regions that are in the G2/M and S phases (Figure 4g).

Figure 4Chaperonin‐related genes are associated with cell cycle progression and promote tumor cell proliferation. (a) Protein–protein interaction (PPI) network of chaperonin‐related genes visualized using the GeneMinia database. Nodes represent individual proteins, and edge colors indicate the types of interaction evidence, including shared protein domains, predicted interactions, physical interactions, pathways, coexpression, colocalization, and genetic interactions. (b) Gene Ontology (GO) and (c) Kyoto Encyclopedia of Genes and Genomes (KEGG) pathway enrichment analyses of chaperonin‐related genes. The number of enriched genes per term is shown as bar length. (d–f) Single‐cell transcriptomic profiling of malignant cells reveals the spatial distribution of chaperonin‐related gene activity in relation to cell cycle states. Single‐cell transcriptomic analysis reveals the expression patterns of chaperonin‐related genes across different cell cycle phases. (d) Chaperonin expression is enriched in specific cell clusters. (e) G2/M phase cells show partial overlap with chaperonin‐expressing populations, suggesting a potential involvement in mitotic regulation. (f) Similarly, S phase cells also exhibit colocalization with chaperonin‐high regions, indicating a role in DNA replication or cell cycle progression. (g) Spatial distribution of cell cycle phases (G1, S, and G2/M) and *MKI67* expression were visualized across multiple HCC tissue sections. Chaperonin expression levels were significantly higher in cells at S and G2/M phases, as shown by violin plots. Spatial heatmaps of *MKI67* expression confirmed proliferative regions, which largely overlapped with areas enriched for chaperonin‐high cells. (h) Western blot analysis confirmed efficient knockdown of *CCT6B* in Huh‐7 and SK‐Hep‐1 cells following transfection with three independent siRNAs. Quantification of band intensity showed a significant reduction in CCT6B protein levels compared to negative control (NC) and untreated cells. (i) Cell proliferation was assessed using the CCK‐8 assay. *CCT6B* knockdown markedly inhibited cell growth over 48 h in both Huh‐7 and SK‐Hep‐1 cells. (j) Flow cytometry analysis of DNA content revealed that *CCT6B* silencing led to cell cycle arrest. In both cell lines, si‐CCT6B induced a significant increase in the G0/G1 phase population and a concomitant reduction in S and G2/M phases.

**Figure:**
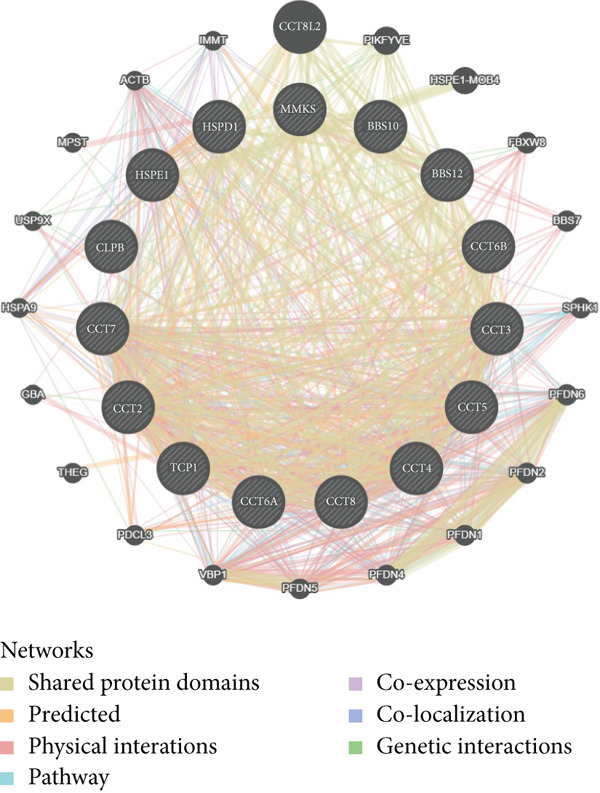
(a)

**Figure:**
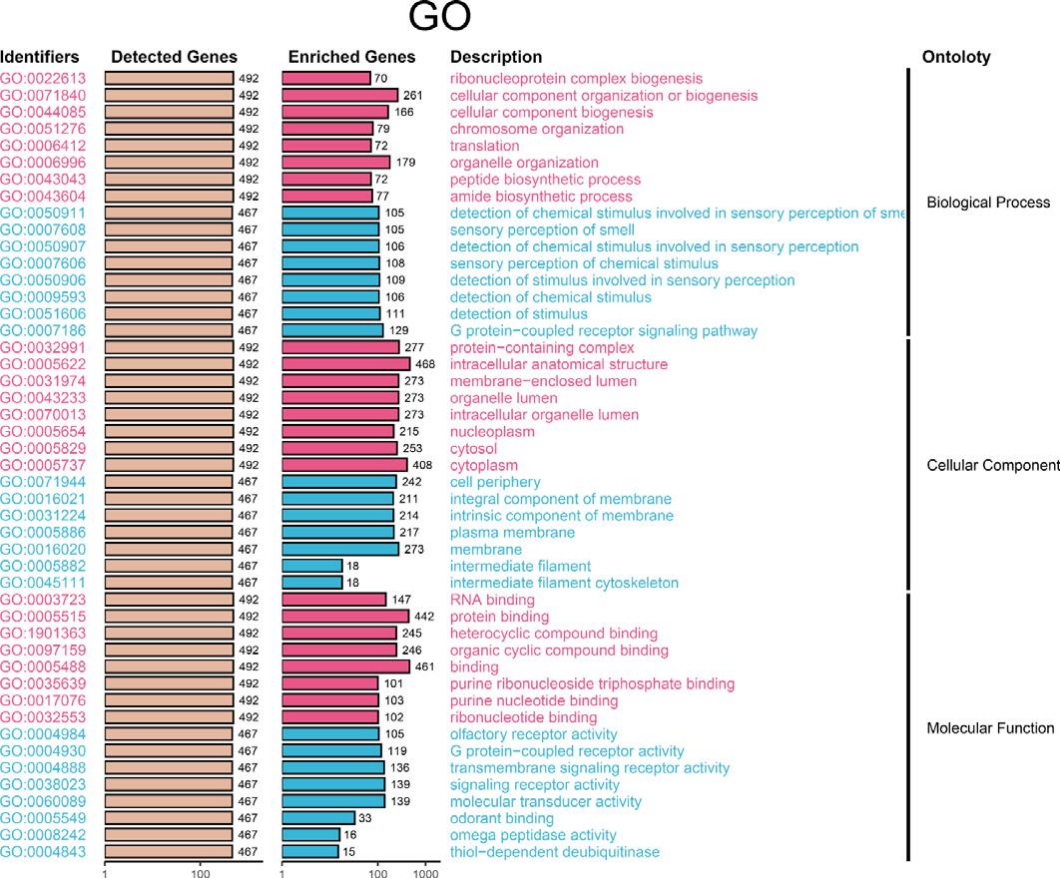
(b)

**Figure:**
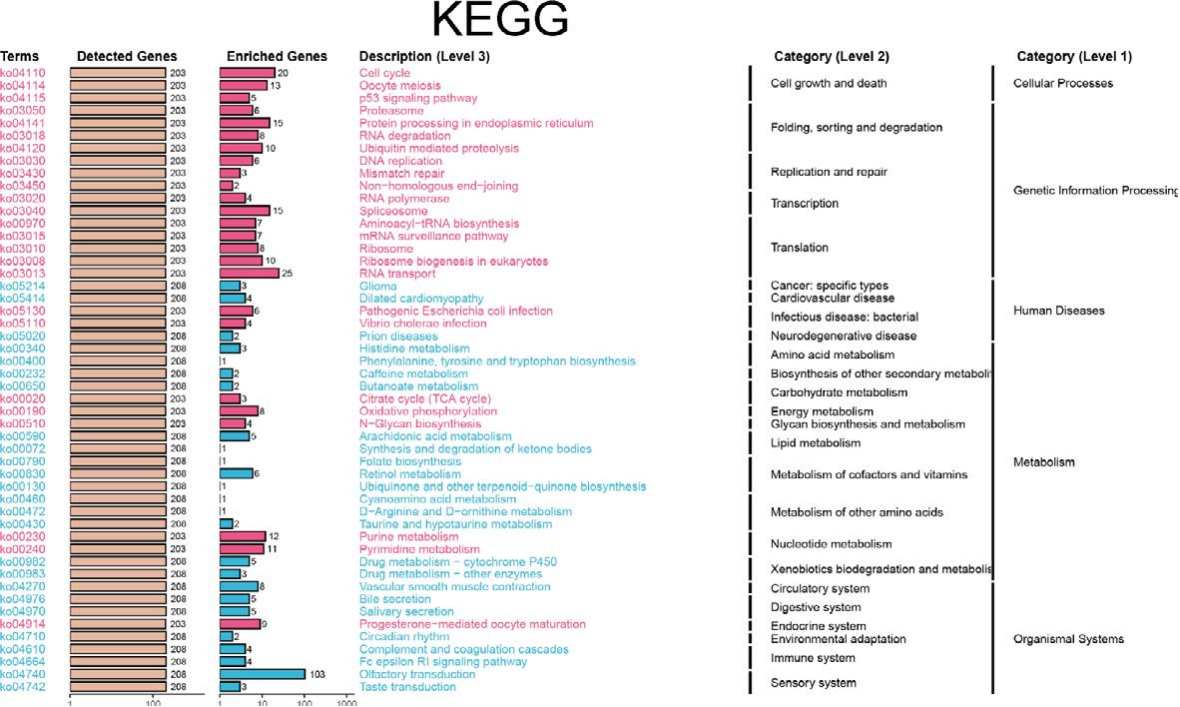
(c)

**Figure:**
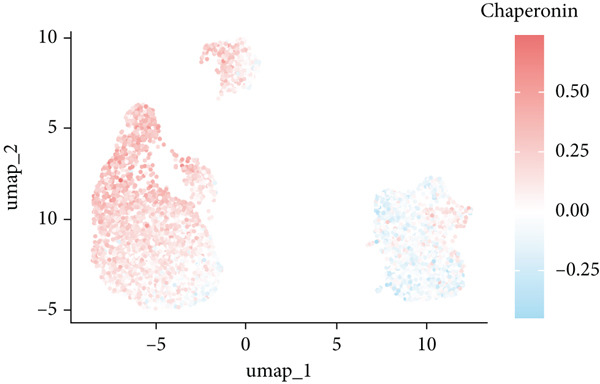
(d)

**Figure:**
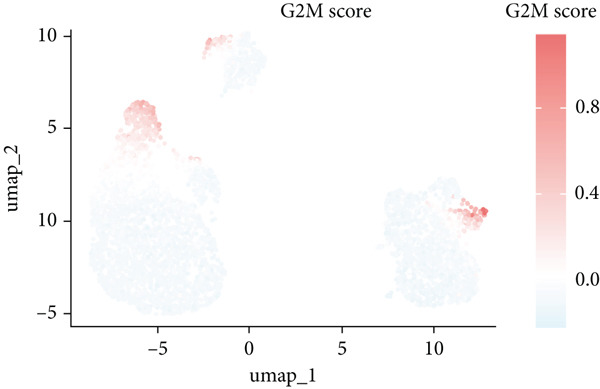
(e)

**Figure:**
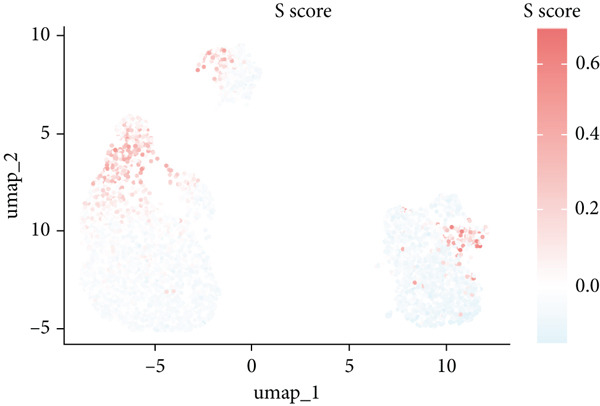
(f)

**Figure:**
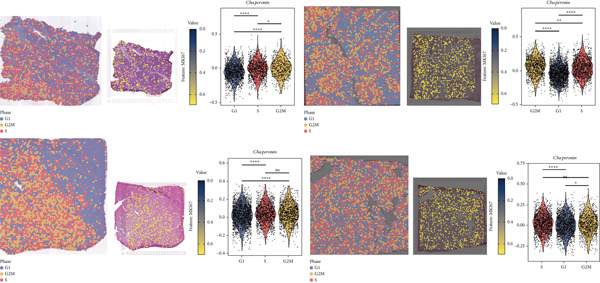
(g)

**Figure:**
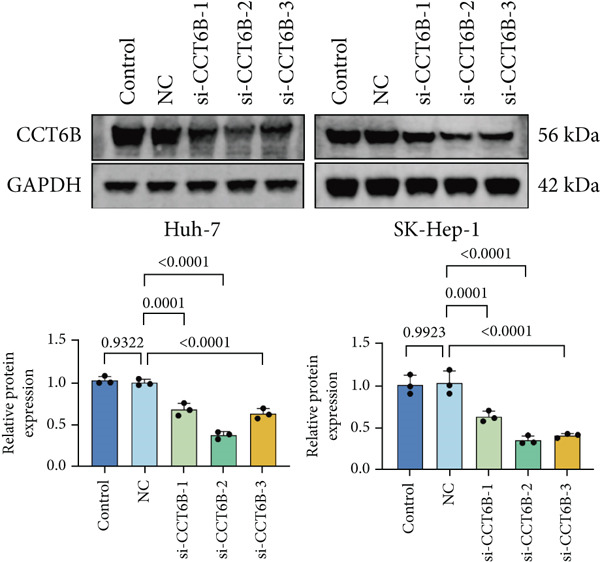
(h)

**Figure:**
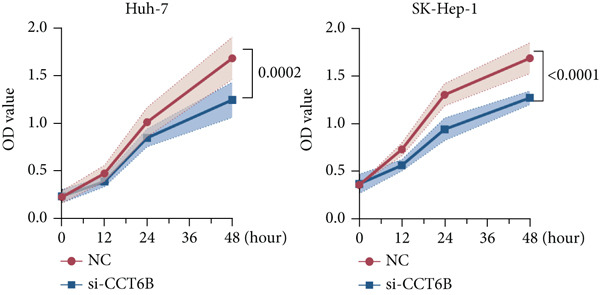
(i)

**Figure:**
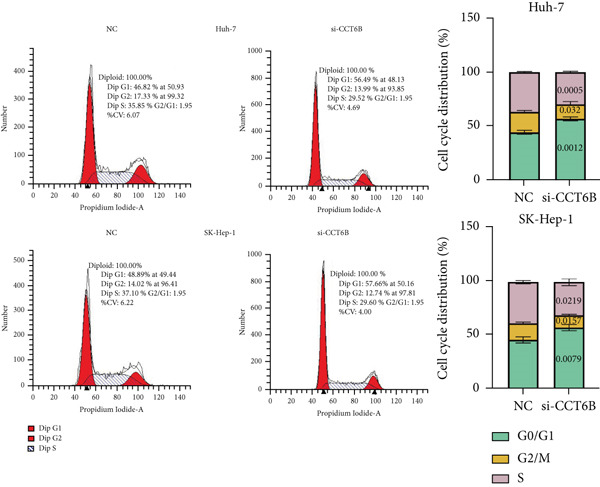
(j)

We next performed experiments to confirm the regulation of the HCC cell cycle by chaperonins. Considering that some of the members of chaperonins have been shown to activate the cycle, we chose *CCT6B*, a previously less studied gene, as the main target in our subsequent study. We designed three siRNAs to knock down *CCT6B* in Huh‐7 and SK‐Hep‐1 cells based on three different target fragment sequences, and the knockdown efficiency was then validated. As shown in Figure 4h, si‐CTT6B‐2 was used for further studies due to its strongest interference effect. We detected the influence of CCT6B on cell proliferation and the cell cycle. The results of CCK‐8 suggested that the knockdown of *CCT6B* suppressed the proliferation (Figure 4i) and induced G0/G1 arrest (Figure 4j) in Huh‐7 and SK‐Hep‐1 cells.

### 3.5. Chaperonins Mediate M2 Macrophage Infiltration

Tumor cells play a pivotal role in orchestrating the development of an immunosuppressive microenvironment by recruiting and activating various immune cells, notably regulatory T cells and M2‐polarized macrophages, which collectively suppress antitumor immune responses [23, 24]. Now that chaperonins have been shown to maintain the malignant phenotype of tumor cells, we next further investigated the relationship between chaperonins and TME. For this purpose, 11 HCC datasets were first utilized to calculate the relationship between CCGs (GSVA score) and the immune microenvironment. The results showed a significant positive correlation between macrophage infiltration levels and CCG expression in most of the datasets (Figure 5a). Importantly, the chemokine CCL20 exhibited a broad, strong positive correlation with CCGs (Figure 5b). Emerging evidence suggests that CCL20, a chemokine secreted by tumor cells, facilitates the recruitment and polarization of macrophages toward an M2 phenotype, thereby promoting a protumorigenic and immunosuppressive microenvironment [25, 26]. We therefore hypothesized that chaperonins might be related to the secretion of CCL20 by tumor cells. To test this speculation, we categorized malignant cells into high‐chaperonin and low‐chaperonin groups based on the median level of CCG expression in the scRNA‐seq data. Figure 5c,d shows that high‐chaperonin malignant cells may have a lower cardinality of communication with other cellular components. Ligand–receptor information for signaling exchanges was further extracted, and as shown in Figure 5e,f, malignant cells high in chaperonin may interact with monocytes/macrophages more frequently via CCL20–CCR6 signaling. We then used the ST data to detect the positional relationship among *CCL20*, *CCR6*, CCGs, and M2 macrophage (Figures 5g, 5h, 5i, and 5j). The analysis revealed distinct clusters wherein CCGs and M2 macrophages exhibited significant colocalization, particularly in regions characterized by robust CCL20–CCR6 signaling activity. Furthermore, we confirm our bioinformatics results in an ex vivo coculture model (Figure 5k). The results of immunofluorescence indicated that the knockdown of *CTT6B* on HCC cells decreased the expression of M2 markers CD163 and CD206 on macrophages (Figure 5l). The results suggest that chaperonins may be a positive upstream factor for M2 macrophage infiltration.

Figure 5Chaperonin programs immunosuppressive macrophage polarization in the tumor microenvironment. (a) Heatmap showing the correlations between CCGs and the relative abundance of immune cell subpopulations in different sample groups, as estimated by multiple deconvolution algorithms (EPIC, CIBERSORT, xCell, MCPcounter, quanTIseq, and TIMER). Each row represents a specific immune or stromal cell type, and columns represent different datasets. Red and blue indicate higher and lower inferred correlations, respectively. (b) Heatmap of the correlations between CCGs and the expression levels of immune‐related genes categorized into antigen presentation (red), immune inhibitors (blue), immune stimulators (orange), chemokines (green), and receptors (purple). (c) Chord diagram illustrating ligand–receptor interactions between various cell types. Lines represent predicted interactions, connecting ligand‐expressing (left half) and receptor‐expressing (right half) cell populations. (d) Heatmap displaying the number of predicted ongoing communications (cardinality) between cell type pairs. Higher cardinality values (yellow) indicate stronger or more frequent interactions. Chaperonin‐high and chaperonin‐low cells showed substantial communication with epithelial cells, fibroblasts, and monocytes/macrophages, indicating their involvement in a broad intercellular signaling network. Dot plots display predicted ligand–receptor interactions from (e) chaperonin‐low and (f) chaperonin‐high malignant cells to tumor‐associated monocytes/macrophages. Dot size indicates intercellular communication strength, and color denotes intracellular signaling activity. Chaperonin‐high cells exhibit enriched signaling through CCL20–CCR6 (circled in red), indicating distinct communication with immunosuppressive or alternatively activated macrophages. (g–j) Spatial transcriptomics (ST) data from four tumor samples were used to assess the positional relationship among *CCL20*, *CCR6*, CCGs, and M2 macrophage signatures. Each panel displays spatial expression patterns overlaid on HE‐stained tissue, with yellow indicating higher expression. Red boxes mark representative regions showing colocalization of *CCL20* and *CCR6* with high levels of CCGs and M2 macrophage markers. These spatial regions suggest that chaperonin‐high malignant cells may contribute to the recruitment or polarization of immunosuppressive macrophages via CCL20–CCR6 signaling within the tumor microenvironment. (k) Schematic of the in vitro induction of M2‐like macrophages. (l) Representative immunofluorescence images showing the expression of M2 macrophage markers CD163 (green) and CD206 (red) in THP‐1‐derived macrophages after coculture with Huh‐7 or SK‐Hep‐1 cells transfected with control (NC) or si‐CCT6B. Nuclei were stained with DAPI (blue). Bar = 20 * μ*m. Quantification of mean fluorescence intensity (right) indicates significantly reduced expression of CD163 and CD206 in macrophages cocultured with si‐CCT6B knockdown HCC cells, suggesting that CCT6B is involved in promoting M2‐like polarization. Data are presented as mean ± SD. Statistical significance was determined using an unpaired two‐tailed Student′s *t*‐test.

**Figure:**
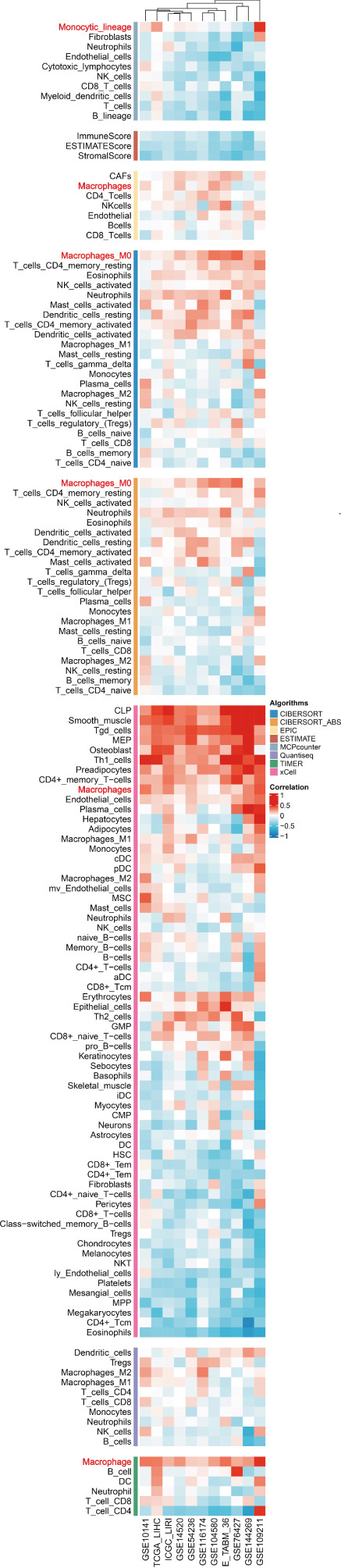
(a)

**Figure:**
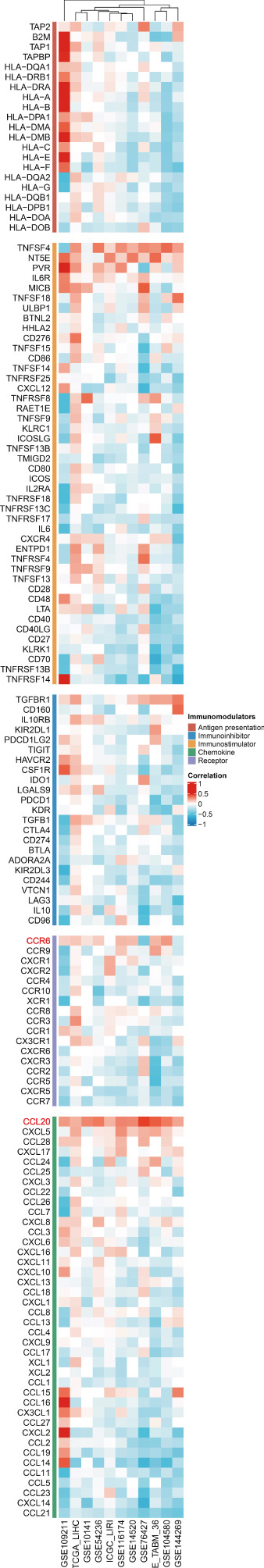
(b)

**Figure:**
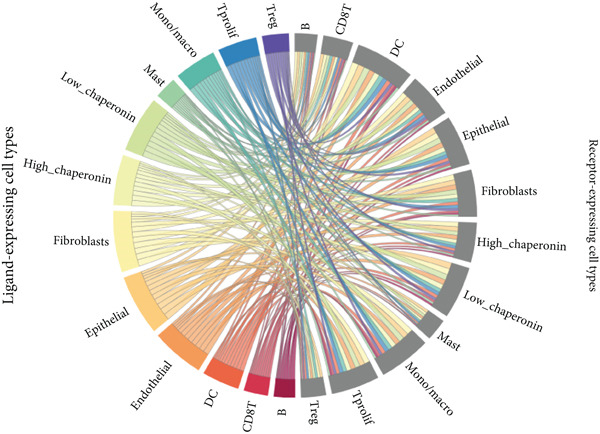
(c)

**Figure:**
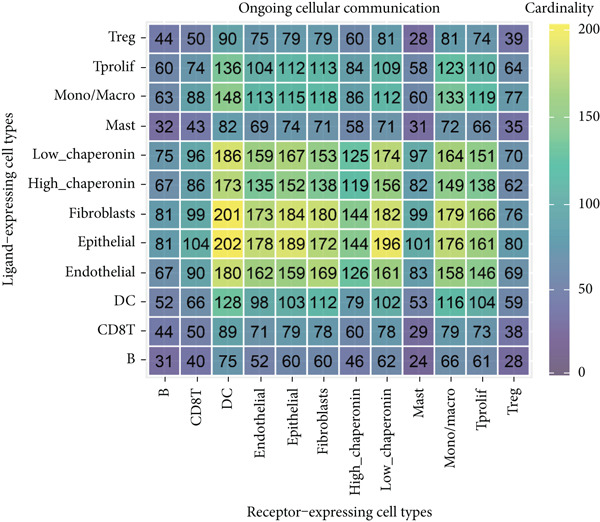
(d)

**Figure:**
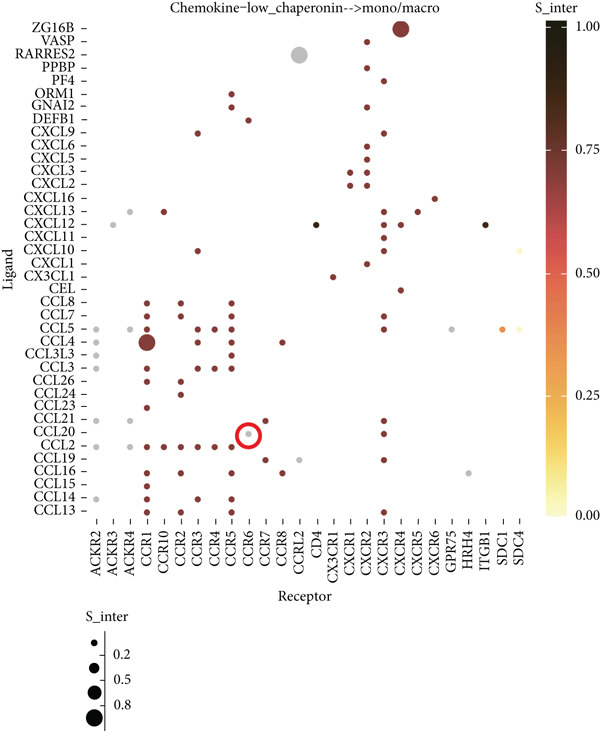
(e)

**Figure:**
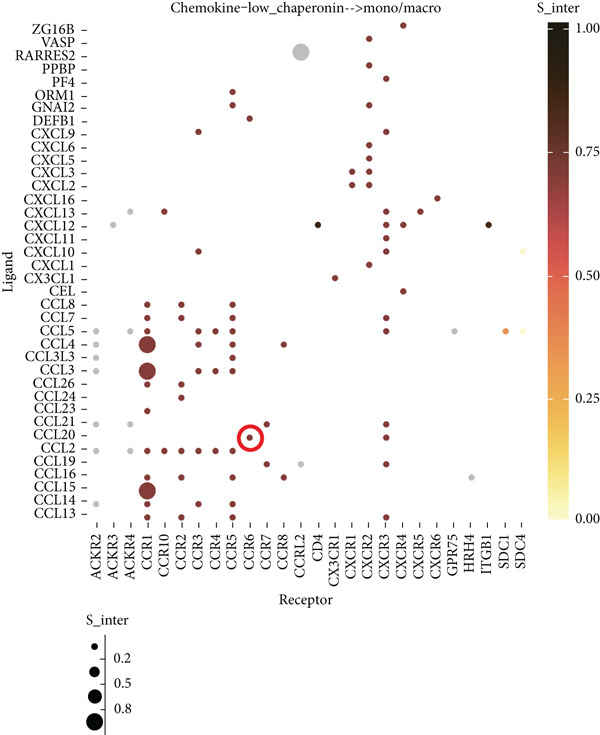
(f)

**Figure:**
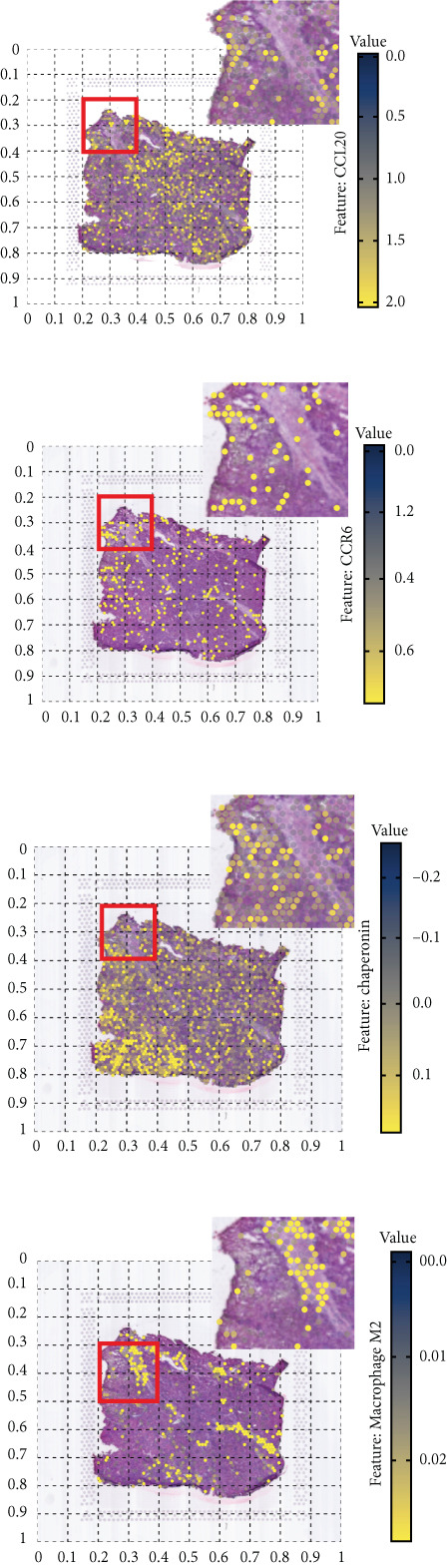
(g)

**Figure:**
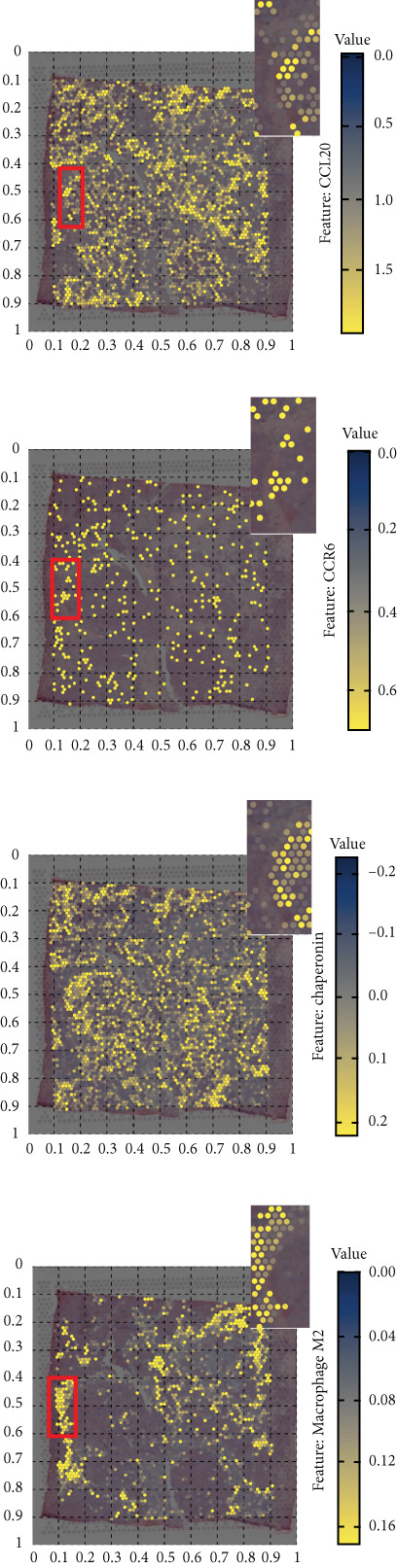
(h)

**Figure:**
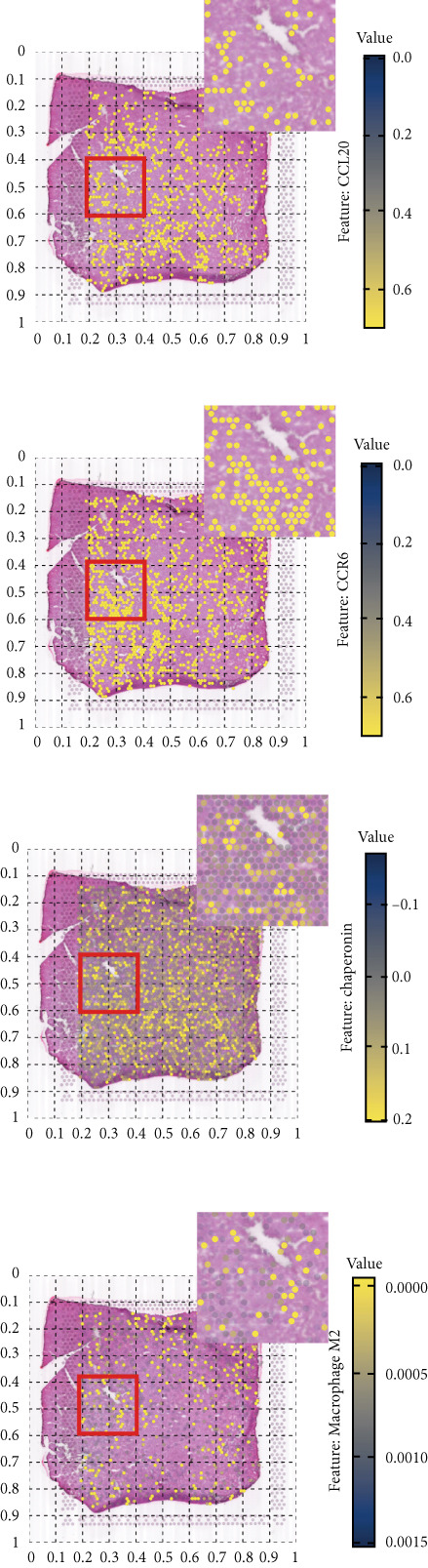
(i)

**Figure:**
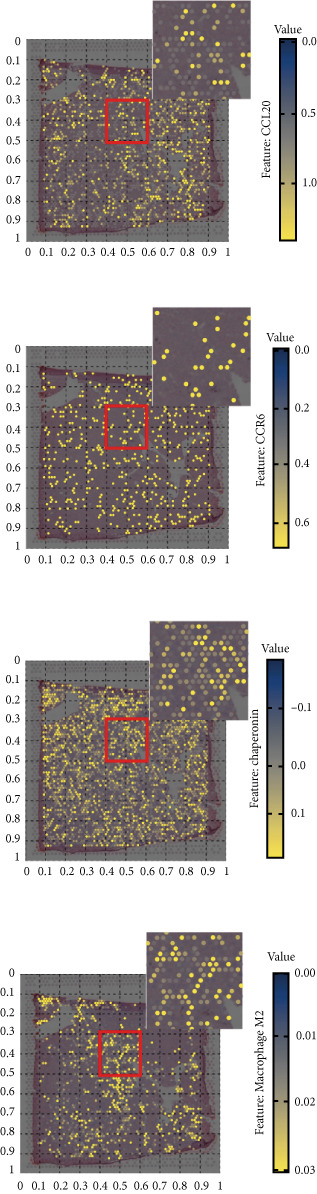
(j)

**Figure:**
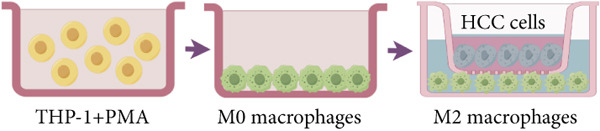
(k)

**Figure:**
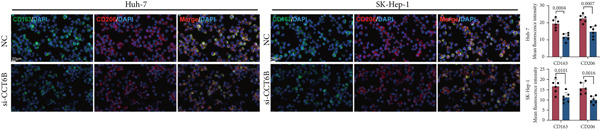
(l)

### 3.6. Chaperonins Are Dependent on *β*‐Catenin Expression

Here, we investigate the causes for the upregulation of chaperonins in HCC. Genomic profiling of the TCGA–LIHC cohort demonstrated significantly elevated rates of somatic mutations, copy number gains, and losses in the high‐chaperonin group relative to the low‐chaperonin group (Figure 6a). Of particular note, *CTNNB1* mutations occurred with statistically greater frequency in tumors with high chaperonin expression (*p* < 0.05). Emerging evidence indicates that *CTNNB1* mutations can initiate oncogenic signaling cascades in HCC by promoting the nuclear accumulation of *β*‐catenin and subsequent activation of Wnt target genes [27, 28]. This dysregulation drives hepatocellular proliferation, survival, and metastasis, underscoring the critical role of *CTNNB1* alterations in hepatocarcinogenesis [29, 30]. The high degree of colocalization of CTNNB1 and CCGs in the ST data from four tumor samples further implies a strong association between the two (Figure 6b). We therefore speculate that the upregulation of chaperonins may be related to *CTNNB1*. To verify this, gene perturbation information about *CTNNB1* in GBSAdb was retrieved, and as shown in Figure 6c,d, the knockdown of *CTNNB1* resulted in the downregulation of G2/M checkpoint signaling in HepG2 cells. We then compared the effect of *CTNNB1* knockdown on the transcriptional levels of CCGs and found that the expression of most CCGs was downregulated by CTNNB1 knockdown, except for *CLPB*, *BBS12*, and *MKKS* (Figure 6e). WB experiments further confirmed this result in Huh‐7 and SK‐Hep‐1 cells (Figure 6f). Thus, the expression of chaperonins may be dependent on *CTNNB1*.

Figure 6Association between chaperonin expression and CTNNB1 (*β*‐catenin) alterations in HCC. (a) Genomic profiling of HCC reveals higher frequencies of *CTNNB1* mutations and copy number gains in tumors exhibiting elevated chaperonin expression. The data illustrate that *CTNNB1* mutations occur with significantly greater frequency in high chaperonin expression tumors (*p* < 0.05), which may contribute to the up‐regulation of chaperonins in HCC. (b) Spatial transcriptomics analysis of four HCC samples showing strong spatial colocalization between *CTNNB1* and CCGs. (c) Volcano plot depicting differentially expressed genes upon *CTNNB1* knockdown in HepG2 cells, based on GBSAdb gene perturbation data. (d) Pathway enrichment analysis of genes downregulated by *CTNNB1* knockdown, highlighting significant suppression of the G2/M checkpoint pathway and other cell cycle–related processes. (e) Transcriptional profiling of CCGs following *CTNNB1* knockdown. Most CCGs showed reduced expression, except for *CLPB*, *BBS12*, and *MKKS*. (f) Western blot validation of chaperonin protein expression in Huh‐7 and SK‐Hep‐1 cells after *CTNNB1* knockdown (*β*‐cat short for *β*‐catenin). Band intensity was quantified and is shown as mean ± SD from three independent experiments. Statistical significance was determined using an unpaired two‐tailed Student′s *t*‐test.

**Figure:**
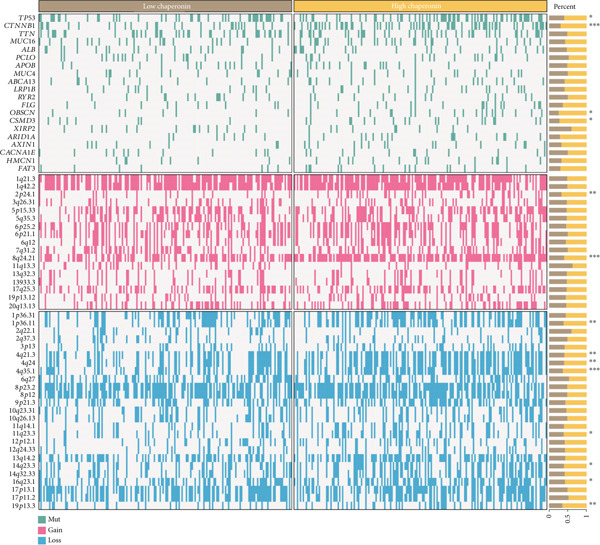
(a)

**Figure:**
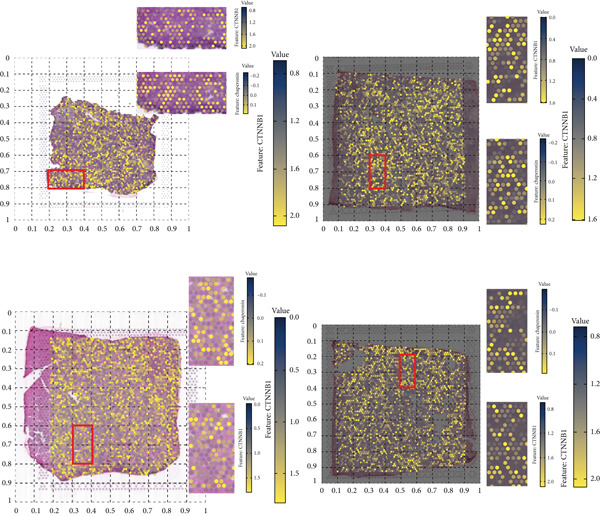
(b)

**Figure:**
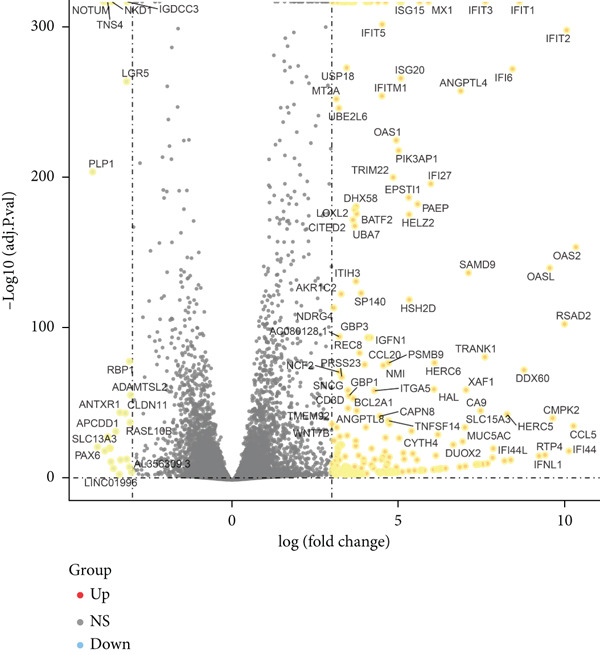
(c)

**Figure:**
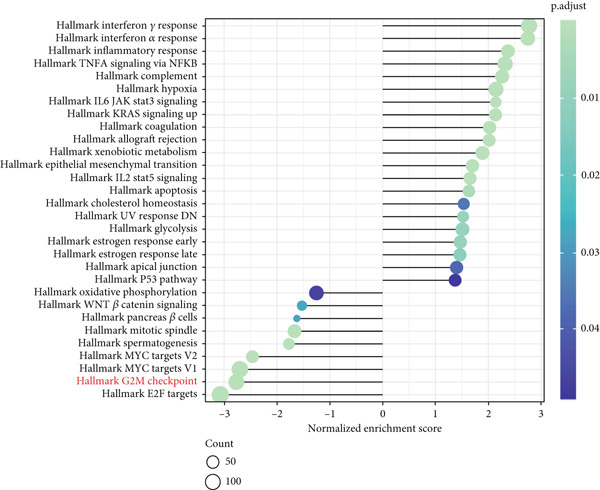
(d)

**Figure:**
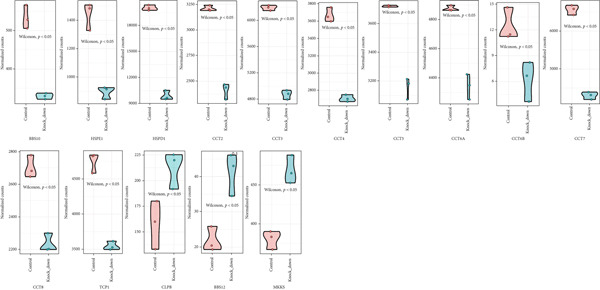
(e)

**Figure:**
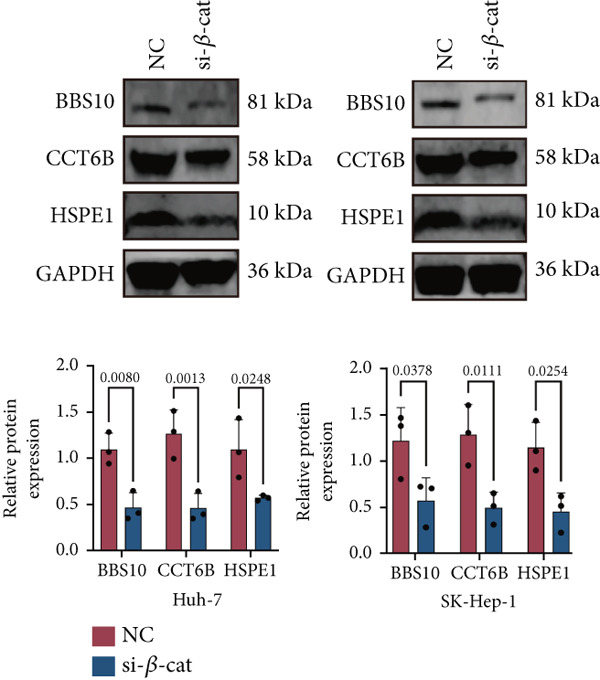
(f)

## 4. Discussion

Chaperonins have emerged as critical players in the intricate landscape of tumor biology [31, 32]. Their primary function, to maintain protein homeostasis by assisting in the folding and refolding of proteins, is particularly crucial in the TME, where oncogenic signaling pathways are often dysregulated [33, 34]. In recent years, a substantial body of research has highlighted the role of chaperonins in sustaining the stability and activity of oncoproteins, thereby promoting tumor growth and survival [35, 36]. This dual role of chaperonins as both facilitators of oncogenesis and potential targets for immunotherapy underscores their importance in cancer research [37–39]. Although chaperonins have been widely studied in various tumor types, many of their mechanisms in HCC remain to be explored [40–42].

Our study builds on this foundation, providing insights into the functional significance of chaperonins in HCC progression. Chaperonins, particularly the CCT subunits, play critical roles in HCC progression [43, 44]. CCT3, CCT6A, and CCT8 are significantly overexpressed in HCC tissues, correlating with poor prognosis, including shorter OS and DFS [32, 45]. CCT3 promotes tumor proliferation, Sorafenib resistance, and ferroptosis sensitivity [41], while CCT6A regulates the G1‐to‐S phase transition, contributing to cell cycle dysregulation and tumor progression [46]. CCT8 is involved in cytoskeletal integrity and tumor invasion, with its depletion inhibiting HCC proliferation.

In addition, TCP1, another chaperonin subunit (CCT7), is significantly upregulated in HCC tissues and correlates with poor prognosis [47]. TCP1 promotes tumor migration via the Wnt7b/*β*‐catenin signaling pathway, enhancing cell proliferation [48]. Collectively, these findings emphasize CCT subunits as important prognostic biomarkers and therapeutic targets, offering new avenues to overcome treatment resistance and improve targeted therapies in HCC.

Our findings indicate that chaperonins are significantly overexpressed in HCC tissues and malignant cells, and this upregulation is associated with more unfavorable survival outcomes. Functional enrichment analyses revealed that chaperonins are involved in numerous signaling pathways, with a particular emphasis on cell cycle regulation. This observation is in line with previous studies demonstrating the ability of chaperonins to stabilize cyclin‐dependent kinases and other mitotic regulators, thereby facilitating unscheduled cell division and tumor progression. Our in vitro experiments further confirmed this by demonstrating that the knockdown of a specific chaperonin, *CCT6B*, suppressed cell proliferation and induced G0/G1 arrest in HCC cell lines. These results collectively suggest that chaperonins play a pivotal role in regulating the cell cycle and promoting tumor cell proliferation in HCC.

The TME is characterized by a complex interplay between tumor cells and various immune cells, including regulatory T cells and M2‐polarized macrophages [49, 50]. Our study found that chaperonins are positively correlated with the infiltration levels of M2 macrophages in HCC tissues. Mechanistically, we hypothesize that this relationship is mediated by the chemokine CCL20, which is secreted by tumor cells and facilitates the recruitment and polarization of macrophages toward an M2 phenotype. Our bioinformatics analysis and ex vivo coculture model provided strong evidence to support this hypothesis, revealing that high‐chaperonin malignant cells interacted more frequently with monocytes/macrophages via CCL20–CCR6 signaling. Furthermore, the knockdown of CCT6B in HCC cells reduced the expression of M2 markers on macrophages, further confirming the role of chaperonins in modulating M2 macrophage infiltration.

The biological drivers of chaperonin upregulation in HCC are only partly understood. Our study suggests that the expression of chaperonins may be dependent on CTNNB1, the gene encoding *β*‐catenin. Genomic profiling of HCC samples revealed a higher frequency of CTNNB1 mutations and copy number gains in tumors with high chaperonin expression. Furthermore, the knockdown of *CTNNB1* resulted in the downregulation of most chaperonin genes, except for *BBS10*, *BBS12*, and *MKKS.* These findings are consistent with previous studies demonstrating the critical role of *β*‐catenin in driving hepatocellular proliferation, survival, and metastasis. Thus, we speculate that CTNNB1 mutations may initiate oncogenic signaling cascades in HCC by promoting the nuclear accumulation of *β*‐catenin and subsequent activation of Wnt target genes, including those encoding chaperonins. Further investigation into the molecular mechanisms underlying this relationship is warranted to fully elucidate the role of chaperonins in HCC pathogenesis.

Despite providing novel insights into the role of chaperonins in HCC pathogenesis, our study has several limitations. Notably, the absence of clinical cohorts limits the translational relevance of our findings. Furthermore, the lack of in vivo experiments to validate the functional roles of chaperonins in tumor progression and immune modulation underscores the need for future studies to address these gaps. These limitations highlight the preliminary nature of our findings and emphasize the necessity for further investigation to fully elucidate the clinical significance and therapeutic potential of targeting chaperonins in HCC.

## 5. Conclusion

In summation, our findings suggest a significant association between chaperonin expression and the malignant phenotype of HCC, offering a potential avenue for further exploration into the therapeutic modulation of these proteins. While additional studies are warranted to fully elucidate the mechanistic underpinnings and clinical implications, the current work underscores the importance of chaperonins as a previously underappreciated factor in HCC pathogenesis. These observations may pave the way for the development of novel diagnostic and therapeutic strategies tailored to the molecular characteristics of individual tumors.

## Ethics Statement

The study′s protocol was approved by the ethics committee of Xiangshui County People′s Hospital (ethics approval number: 3‐23‐10RO; ethics approval date: 2023‐10‐13).

## Consent

Prior to the commencement of data collection, the first author obtained written consent from all participants concerning participation and subsequent publication of the study results.

## Disclosure

All authors contributed to data analysis, drafting, or revising of the article, agree on the journal to which the article is being submitted, provided final approval of the version to be published, and agree to be accountable for all aspects of the work.

## Conflicts of Interest

The authors declare no conflicts of interest.

## Author Contributions

Shou‐hua Wang and Feng‐ya Lv drafted the manuscript, Yuan‐jie Liu and Jie‐pin Li performed all analyses, Jia‐qi Hao provided the site for this study, and Hong‐hua Wang provided the research funding and reviewed the manuscript. Shou‐hua Wang and Feng‐ya Lv contributed equally to this study.

## Funding

This study was funded by the Medical Research Projects of Yancheng City Health Commission, YK2023101 and YK2023102.

## Supporting information


**Supporting Information** Additional supporting information can be found online in the Supporting Information section. Chaperonin‐related genes included in this study, as curated from the HGNC database (Table S1).

## Data Availability

The data that support the findings of this study are available from the corresponding author upon reasonable request (Hong‐hua Wang: xswhh@sina.com).
